# Analysis of Whole-Brain Resting-State fMRI Data Using Hierarchical Clustering Approach

**DOI:** 10.1371/journal.pone.0076315

**Published:** 2013-10-18

**Authors:** Yanlu Wang, Tie-Qiang Li

**Affiliations:** 1 Department of Clinical Sciences, Intervention, and Technology, Karolinska Institute, Stockholm, Sweden; 2 Department of Medical Physics, Karolinska University Hospital, Huddinge, Sweden; University of Maryland, College Park, United States of America

## Abstract

**Background:**

Previous studies using hierarchical clustering approach to analyze resting-state fMRI data were limited to a few slices or regions-of-interest (ROIs) after substantial data reduction.

**Purpose:**

To develop a framework that can perform voxel-wise hierarchical clustering of whole-brain resting-state fMRI data from a group of subjects.

**Materials and Methods:**

Resting-state fMRI measurements were conducted for 86 adult subjects using a single-shot echo-planar imaging (EPI) technique. After pre-processing and co-registration to a standard template, pair-wise cross-correlation coefficients (CC) were calculated for all voxels inside the brain and translated into absolute Pearson's distances after imposing a threshold CC≥0.3. The group averages of the Pearson's distances were then used to perform hierarchical clustering with the developed framework, which entails gray matter masking and an iterative scheme to analyze the dendrogram.

**Results:**

With the hierarchical clustering framework, we identified most of the functional connectivity networks reported previously in the literature, such as the motor, sensory, visual, memory, and the default-mode functional networks (DMN). Furthermore, the DMN and visual system were split into their corresponding hierarchical sub-networks.

**Conclusion:**

It is feasible to use the proposed hierarchical clustering scheme for voxel-wise analysis of whole-brain resting-state fMRI data. The hierarchical clustering result not only confirmed generally the finding in functional connectivity networks identified previously using other data processing techniques, such as ICA, but also revealed directly the hierarchical structure within the functional connectivity networks.

## Introduction

Taking advantage of the rapidly expanding computational power in the past decade, several studies showed the feasibility to analyze whole-brain resting-state fMRI data with clustering algorithms. For example, Benjaminsson et al. used a dimensional scaling and vector quantization clustering technique to analyze resting-state fMRI data [1]. Van den Heuvel et al. used a graph-theory approach to determine several functional connectivity networks [2].

Hierarchical clustering has not been used as fluently as other clustering methods in the analysis of resting-state fMRI data due to its poor scaling, high complexity and sensitivity to outliers. On the other hand, hierarchical clustering is completely deterministic and can stratify data into a hierarchical structure [3], [4], [5], [6]. Previous studies on hierarchical clustering of resting-state fMRI data have been limited to a few slices or region-of-interests (ROIs) after substantial data reduction. Cordes et al. used a hierarchical clustering algorithm and analyzed 4 slices of resting-state fMRI data [7]. In another hierarchical clustering study of human brain, Salvador et al. grouped the resting-state fMRI data into regions-of-interests (ROIs) according to their anatomical locations prior to the clustering [8] of the ROIs. More recently, voxel-wise hierarchical clustering was also attempted on resting-state fMRI data acquired from rodents [9].

Due to limited computational capacity, data reduction is usually needed in hierarchical clustering of resting-state fMRI data. In the earlier works this was achieved either by limiting the number of slices [7] or substantially reducing the data into anatomical ROIs [8]. In order to perform voxel-wise hierarchical clustering of whole-brain resting-state fMRI data, in this study we used a brain mask to achieve data reduction without compromising spatial resolution and coverage. This masking operation also improves the robustness of the framework by eliminating irrelevant voxels containing noise and artifact outliers which destabilize the algorithm [10]. To further improve stability of the algorithm, the distance matrix for individual subject was thresholded and averaged prior to the clustering. This allows also efficient group analysis of whole-brain resting-state fMRI data.

We developed an iterative scheme with termination criteria based on cluster size to analyze the dendrogram. With the developed framework, we were able to independently identify most of the functional connectivity networks reported previously in the literature using ICA and other analysis methods. Furthermore, we illustrated that the dendrogram can directly reveal the inherent hierarchical structure within the functional connectivity networks. To the best of our knowledge, there has been no previous study succeeded in hierarchical clustering of whole-brain human resting-state fMRI data at voxel-level.

## Materials and Methods

### Ethical statement

This study was approved by the Central Ethical Review Board in Sweden, who also approved the consent form used to provide information and obtain consent. All participants provided informed consent by signature.

### Data acquisition

Resting-state fMRI measurements were conducted for 86 normal adult subjects (male/female = 40/46, aged 21–84 years old). All resting-state fMRI measurements were performed on a Siemens whole-body 3T clinical MRI scanner (Magnetom Trio, Erlangen, Germany) using a dedicated 32-channel phased array detector. For each subject, at least one set of resting-state fMRI data was acquired using a single-shot 2D gradient-recalled echo (GRE) echo-planar imaging (EPI) technique. The essential acquisition parameters for the resting-state fMRI scan included the following: 32 transverse slices of 3.6 mm thick, TR/TE = 2000/35 ms, FOV = 220 mm, matrix size = 64×64, parallel imaging acquisition with an acceleration factor (IPAT) of 2, flip angle = 90°, and 300 dynamic timeframes.

### Data preprocessing

Data preprocessing were performed using AFNI (http://afni.nimh.nih.gov/afni/) and FSL (http://www.fmrib.ox.ac.uk/fsl) programs wrapped around a bash shell script. The first 10 timeframes in each data set were removed to ensure the signal reaches steady state. Head motion correction was performed based on 6-parameter rigid body images registration. The average volume for each motion corrected time series were used to remove the skull from the images and to create whole-brain mask. Spatial normalization to the MNI template was performed using 12 parameter affine transformation and mutual information as the cost function. The data was then resampled to isotropic resolution using a Gaussian kernel with FWHM = 4 mm. Low-pass filtering at 0.1 Hz was done followed by baseline de-trending up to the third order.

### Cross-correlation evaluation

**Figure 1:**
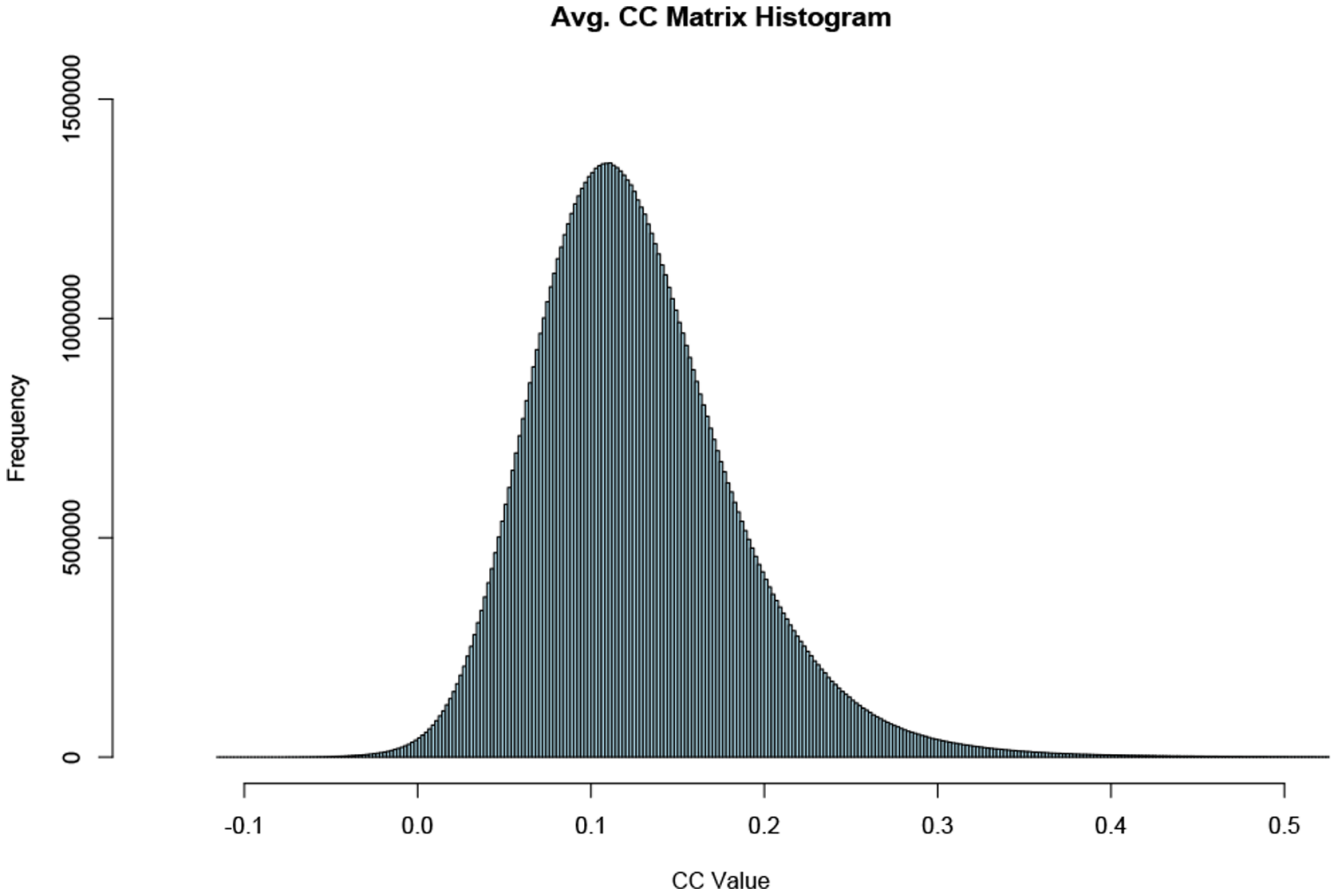
Average CC matrix histogram. Histogram of the average CC matrix of all datasets. The majority of CC values are below 0.2 with a relatively small number of values above 0.4. Negative values exist but are relatively few and only take on small values.

To minimize processing load without promising the spatial resolution and whole-brain coverage, individual datasets were masked with a standard gray matter template derived from FSL tissue priors (http://www.fmrib.ox.ac.uk/fsl) to exclude white matter and cerebral spinal-fluid (CSF) regions. After the masking, the pair-wise Pearson's cross correlation coefficients (CC) were calculated for all datasets. The correlation coefficients were then thresholded at 0.3 [7] The correlation values below the threshold was truncated to zero while values above this threshold were not changed. After thresholding, approximately 1.1% of the correlation coefficients remained for further analysis (Fig. 1). The cross correlation matrices for all subjects were then averaged together voxel-wise. The averaged correlation data were then converted into absolute Pearson's distances according to the definition (1-|CC|), which were used to perform hierarchical clustering.

### Hierarchical clustering

An agglomerative hierarchical clustering algorithm was used as the basis for the framework. A brief description of the algorithm is summarized as follows: Given a set of *N* voxels to be clustered, and a corresponding *N×N* distance matrix:

1. Assign each voxel to a cluster, resulting in *N* clusters, with each cluster containing just one voxel. The distances between the clusters are the distances among the voxels.
2. Find the closest pair of clusters.
3. Merge the closest pair of clusters, resulting in one cluster less in total.
4. Repeat 2–3 until only a single cluster remains.

Step 3 can be performed in a variety of ways, referred to as linkage methods. The type of linkage in a hierarchical clustering algorithm refers to how the algorithm determines distance between newly formed clusters to all other voxels and clusters. Single-linkage takes the shortest distance between new clusters against the rest of the data, maximum-linkage takes the longest distance, and average-linkage takes the average. In our application, voxels within a cluster corresponding to a functional connectivity network should be highly correlated to each other. Hence, single-linkage is not desirable in this application. Maximum-linkage forces the algorithm to solely determine clusters with all voxels having high correlations to each other without exceptions. Average-linkage relaxes somewhat the intra-cluster connectivity requirements compared to maximum-linkage by taking the average distance. Hence, average-linkage was opted to take into account of the potential noise residues. Pseudo-code for the algorithm is shown in Appendix A in Supporting Information S1.

The algorithm produces a binary hierarchy tree (dendrogram) from which *k* clusters can be retrieved by cutting the k-1 longest links.

Since the number of meaningful clusters is unknown *a priori* and is affected by the noise level of the resting-state fMRI data, it is difficult to specify the number of resulting clusters directly. We tested different criterion for the selection of clusters, e.g. the inconsistency coefficient (see Appendix B in Supporting Information S1 and Figure S1 for details). It was found that the cluster size could be used as an effective approach. By referring to the group ICA result from the same dataset, a cluster larger than 5000 voxels in size (S≥5000) is considered too large to be a single functional connectivity network, whereas a cluster less than 50 voxels in size (S≤50) is considered too small to be a meaningful cluster. The selection of these parameters is determined semi-empirically and is discussed in further details below.

**Figure 2:**
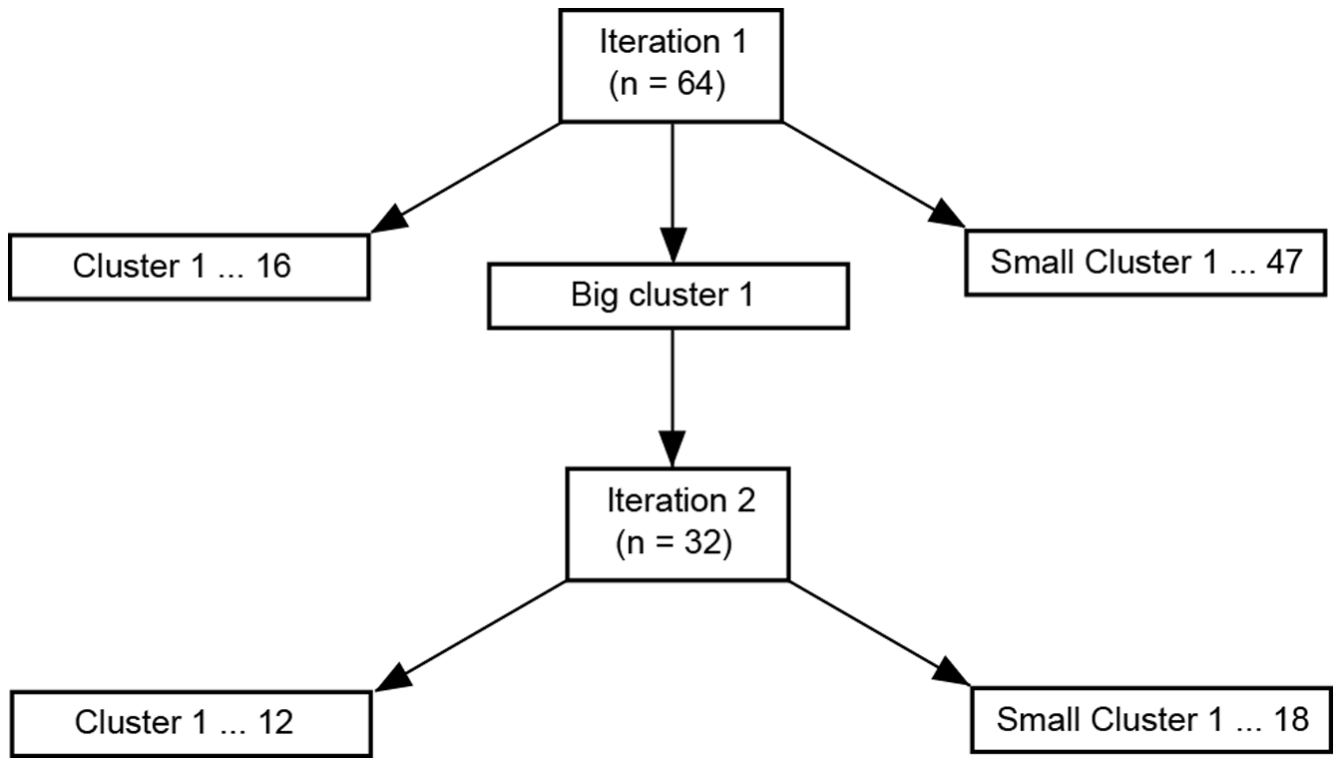
Schematics of the iterative clustering pipeline. Schematics for iterative hierarchical clustering framework based on resulting cluster size. Small clusters are excluded and large clusters are clustered further iteratively using a smaller cluster count.

Testing the algorithm with different number of clusters indicates that increasing the number of clusters in the first iteration simply increases the number of spurious small clusters but does not efficiently reduce the size of the largest cluster, once the cluster count is sufficiently large. Therefore, the cluster count, for the first iteration (whole-brain clustering), was set to 64. Cluster count for the hierarchies further down was reduced by a factor of 2 than that for the previous iteration (*k_2_ = 32, k_3_ = 16, k_4_ = 8 etc.*). The cluster number for subsequent iterations is decreased according to the size of the large cluster to be further divided to avoid spurious amounts of small clusters being generated. An overview of the hierarchical clustering pipeline is schematically shown in Figure 2.

In order to identify potential resting-state functional networks (RFNs), Clusters with adequate voxel size (50≤S≤5000) were carefully examined by comparing their spatial distribution patterns with previously published RFNs in the literatures.

### ICA analysis

In order to verify the RFN results from the hierarchical clustering, independent component analysis (ICA) of the same resting-state fMRI data sets were also performed using the GIFT toolbox, v1.3h (http://www.nitrc.org/projects/gift) implemented in MATLAB (MathWorks, Massachusetts, U.S.A). Individual data set was first concatenated and then followed by computation of the individual ICA components and corresponding time courses. Principle Component Analysis (PCA) was used prior to ICA for data reduction. The InfoMax group-ICA algorithm was then applied on the reduced data. Lastly, back-reconstruction of time series data for each individual subject was performed. The number of predefined ICA components was set to 36, as done in accordance to a previous study [11]. Independent components (ICs) that are common for the entire subject group and resemble RFNs were identified through a threshold of voxel-wise t-maps and visual examination of the spatial distribution patterns.

### Comparison between hierarchical clustering and ICA results

For quantitative comparison between the hierarchical clustering results and RFNs identified by ICA, each cluster that fulfilled the cluster size criteria (50≤S≤5000) was matched with a RFN identified using ICA based on its maximum overlap and the similarity of the spatial distribution. The cluster size, intersection area, and complementary non-overlapping areas were evaluated for each matched pair.

### Assessment of statistical significance

Because the CC matrices were thresholded at 0.3, which corresponds to a t-score threshold of 2.88 (df = 84), the statistical significance at voxel-level is p<0.001 without correcting for multiple comparisons. For comparison the group ICA spatial t-maps were then binarized at voxel-level with the same voxel-level statistical significance.

The final statistical significance was evaluated by enforcing also a minimum voxel cluster size of 6 contiguous voxels. After specifying the original and final voxel sizes as well as the uncorrected threshold value the AFNI program, *AlphaSim*, was used to compute a list of probabilities corresponding to different cluster sizes produced by random field of noise. The final voxel size after pre-processing was 4×4×4 mm^3^. The used interpolation kernel along the slice direction was bicubic. The in-plane blurring kernel was Gaussian function. By enforcing a minimum cluster size of 6 contiguous voxels, probability simulations based on *AlphaSim*, using 10^5^ iterations indicate that the probability of random field of noise producing a cluster of size ≥6 is at p<0.05 after the noise was thresholded at pixel level with p<0.001.

## Results

**Figure 3:**
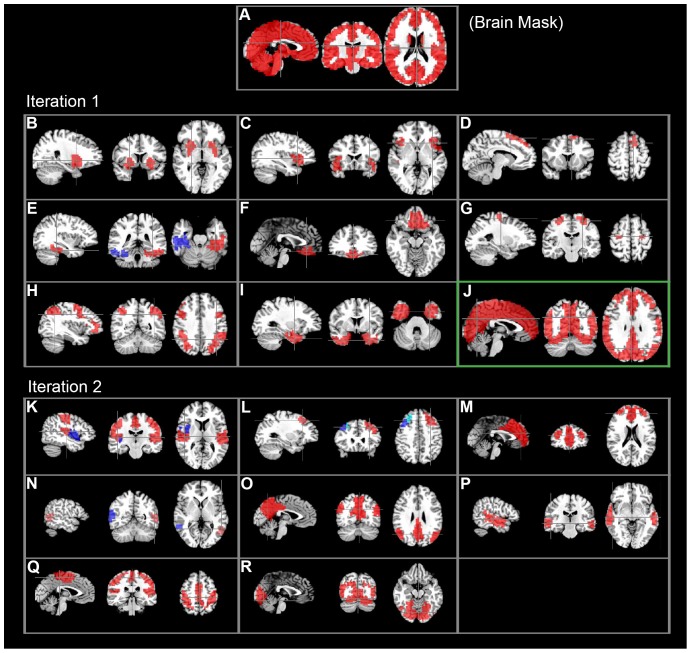
Hierarchical clustering result. Clustering results from the selected cut levels. Bilaterally symmetric clusters are displayed in the same figures using two different colors (blue and red). The large cluster went through further iteration is marked with a green box.

There were 37 out of a total of 112 clusters that fulfilled the cluster size criteria (Table 1). As summarized in Figure 3, a total of 20 out of the 37 clusters were identified as potential RFNs by careful inspection of the spatial patterns. The characteristics of these identified RFNs were summarized in Table 2. The remaining 17 clusters were classified as likely artifacts after studying their spatial distribution patterns. Small clusters with less than 50 voxels usually have single or too few voxels to be considered as meaningful RFNs.

**Table 1:** Cluster count for hierarchical clustering.

Iteration	Big cluster count	Small cluster count	Potential RSN cluster count	Total cluster count
1	1	47	16	64
2	0	18	14	32
Total	1	65	30	96

**Table 2:** Hierarchical clustering results.

Label	Size (Voxels)	Min CC	Max CC	Average CC
B	312	0.17	0.29	0.24
C	330	0.17	0.30	0.24
D	66	0.19	0.30	0.25
E (left/right)	168/176	0.17/0.19	0.25/0.26	0.22/0.22
F	270	0.18	0.29	0.24
G	66	0.19	0.28	0.24
H	1311	0.16	0.27	0.22
I	672	0.17	0.27	0.22
J	6828	0.15	0.29	0.22
K, blue region	240	0.20	0.31	0.27
L (left/right)	189/114	0.21/0.24	0.32/0.34	0.31/0.30
M	888	0.20	0.33	0.27
N (left/right)	67/91	0.25/0.22	0.36/0.34	0.31/0.30
O	999	0.19	0.36	0.29
P	463	0.19	0.30	0.26
Q	1540	0.19	0.35	0.27
R	1619	0.18	0.36	0.29

As shown in Figs. 2 and 3, the first iteration hierarchical clustering produced 8 RFNs (Figs. 3B–I) including two frontal networks (Figs. 3D and F), dorsolateral frontal network (Fig. 3C), premotor network (Fig. 3G) and 3 RFNs involving the temporal cortex. Two of the RFNs detected in the temporal lobes were split into right- and left-sided clusters along the hemisphere middle line, as indicated with two different colors (Figs. 3E, L, and N). This iteration produced also a large cluster with 6828 voxels (Fig. 3J) for further clustering in the second round.

The second iteration of the hierarchical clustering on the large cluster (Fig. 3J) produced 6 commonly observed RFNs including the default mode network (DMN) (Figs. 3M and O), visual network (Fig. 3R), sensorimotor network (Fig. 3K), and 2 temporal networks (Figs. 3N and P). The sensory motor network (Fig. 3K) includes the motor, somatic sensory, parts of the auditory cortex and parietal region. The visual network (Fig. 3R) covers the primary and secondary visual cortices and posterior hippocampus. The second iteration results in more highly intra-connected clusters. As shown in Table 2, the mean of the averaged intra-cluster CC for the second iteration is 0.29 while it is 0.23 for the first iteration.

Analysis of the same dataset with ICA, 13 independent components out of 36 were identified as relevant RFNs based on t-score threshold and visual inspection of the t-maps and corresponding time courses, whereas the remaining 23 components were classified as artifacts due to contamination from CSF, motion, and large veins.

**Figure 4:**
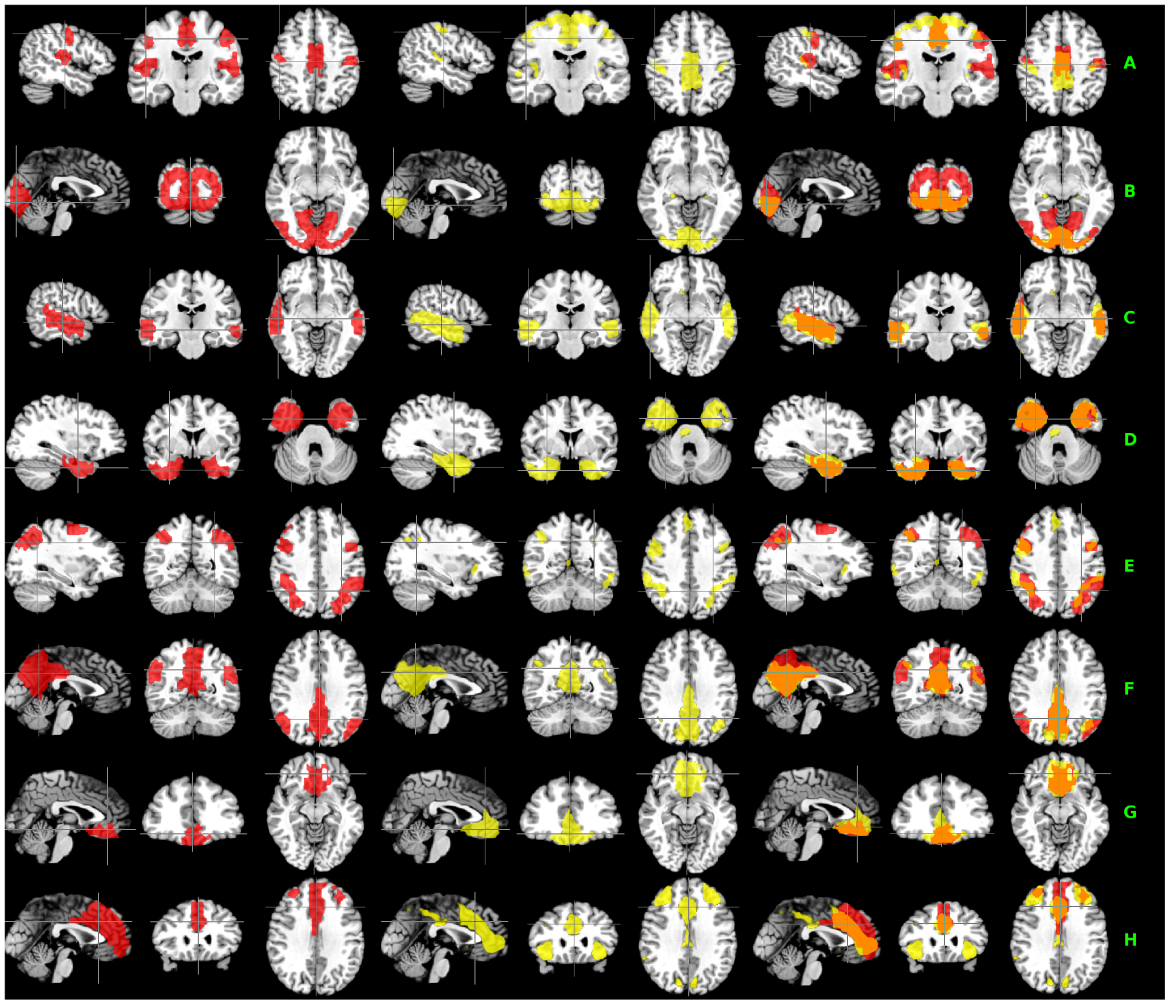
Comparison between the hierarchical clustering and ICA results. Set of hierarchical clusters (*left column*) and ICA components (*middle column*) with good matching. The right column displays their corresponding overlays with overlapping areas showing in orange. The group ICA t-maps were binarized with a threshold at p<0.001 for ease of comparison with clustering results.

Among the extracted 20 potential RFN clusters by using the proposed hierarchical clustering scheme, there are 8 clusters having relatively good match with RFNs identified by ICA. The details are summarized in Figure 4 and Table 3. As shown, the 8 RFN clusters cover about 58% of the total grey matter volume. For these 8 clusters, on average, there is a 61% spatial overlap between the hierarchical clustering and ICA results.

**Table 3:** Comparison between the ICA and clustering results.

Label	Cluster size	Component size	Intersect	Exclusive cluster	Exclusive component
A	1540	1100	636	904	464
B	1619	494	469	1125	25
C	463	841	399	64	442
D	672	843	537	135	306
E	1311	1099	558	753	541
F	999	765	517	482	248
G	270	481	247	23	234
H	888	1550	594	294	956

## Discussion

### Choices of parameters for the hierarchical clustering framework

**Figure 5:**
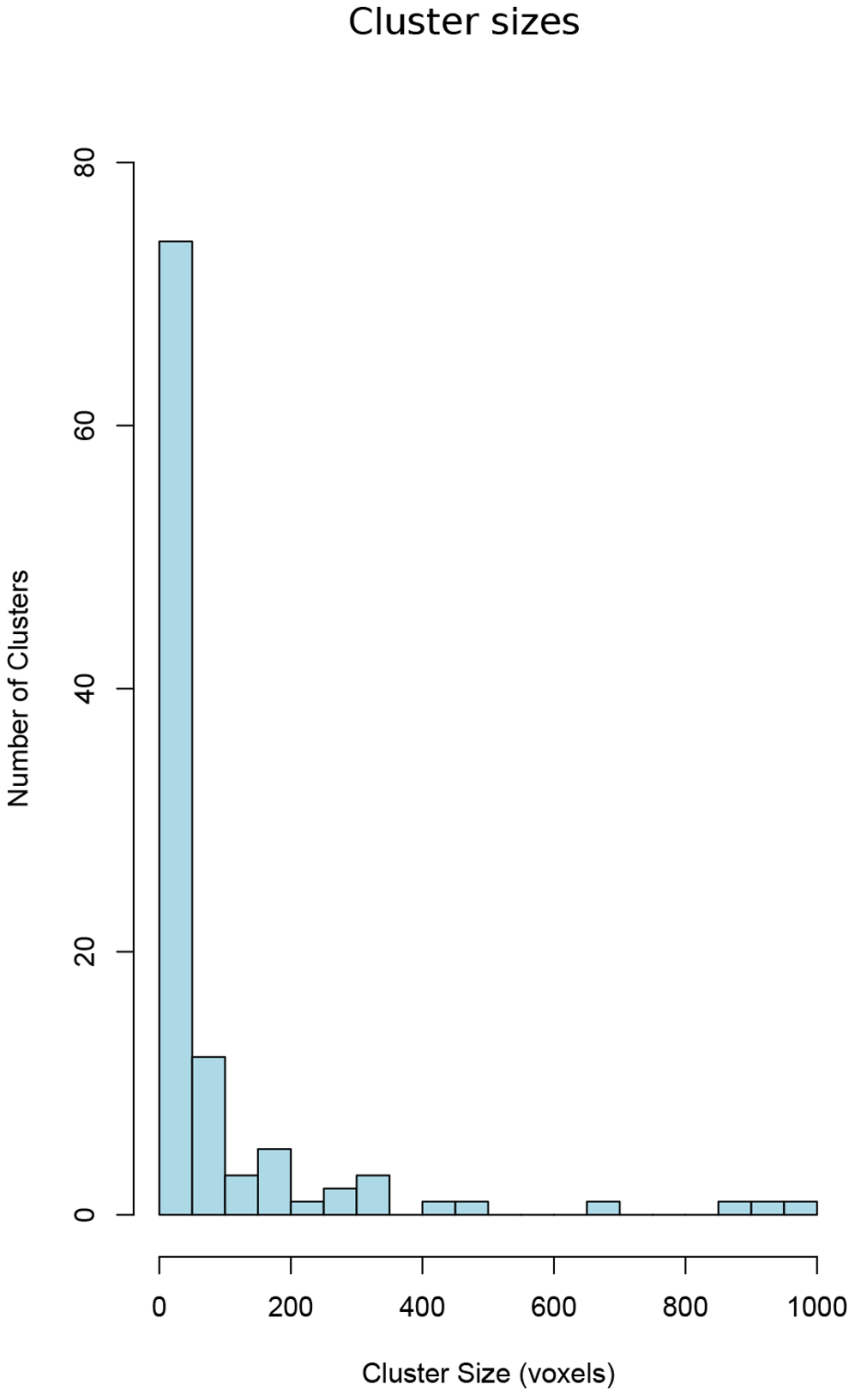
Histogram of cluster size. Cluster size from all clusters produced by applying the proposed framework to the acquired resting-state fMRI datasets. It is clear that many clusters have less than 50 voxels.

**Figure 6:**
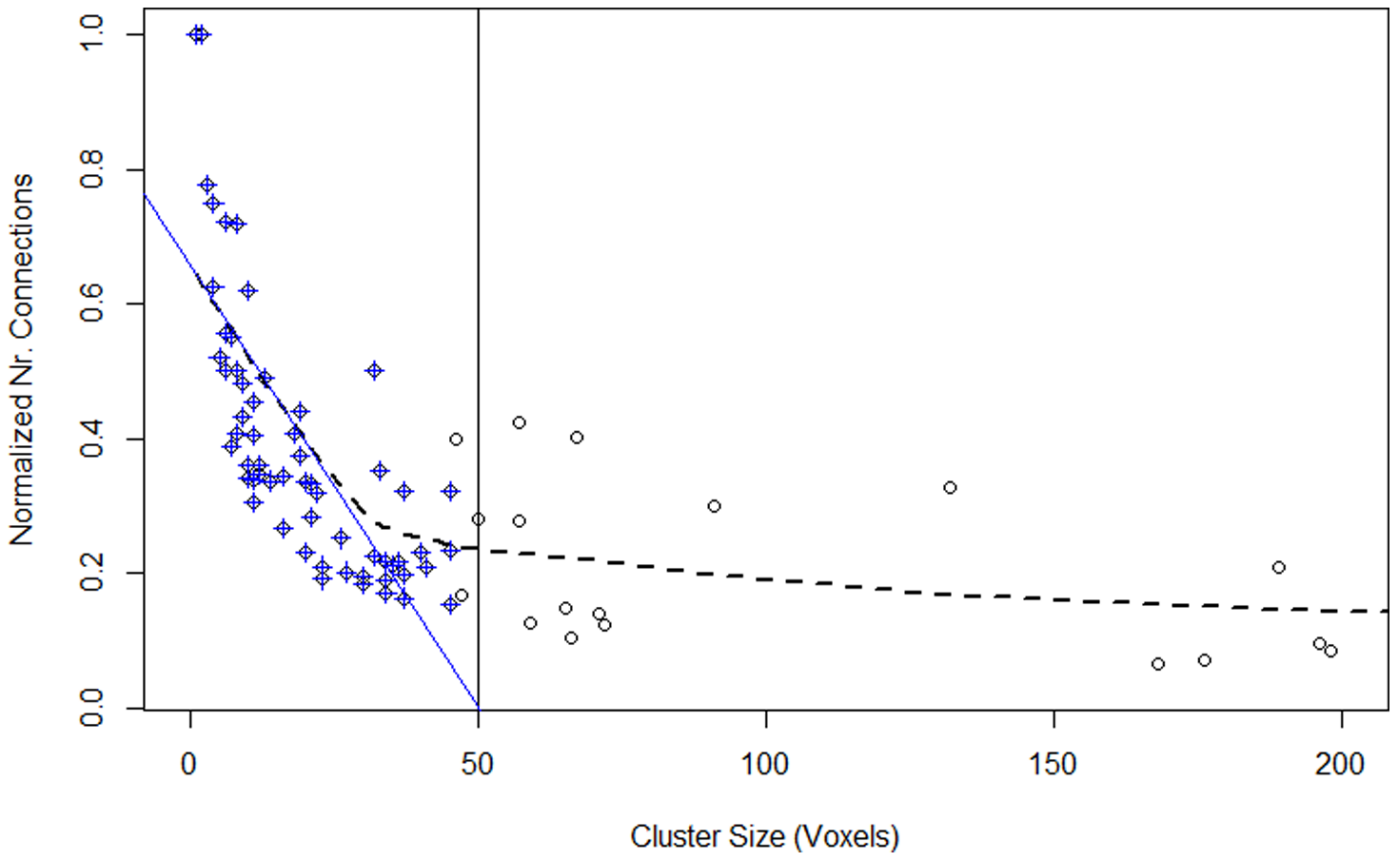
Intra-cluster connections of the clusters. Scatter plot of the normalized intra-cluster connections (the ratio between the number of connections with CC≥0.3 and the number of possible connections) as a function of the cluster size. Locally weighted scatterplot smoothing (LOESS) regression analysis (dashed curve) shows a distinct change in trends in the data. To emphasize this change, linear regression (shown in blue) was used to extrapolate the initial distinct trend of the LOESS curve. Linear regression occupied 63 of the smallest clusters (shown in blue crosses) and intersects the x-axis at 50.02. This observation was used to determine the minimum cluster size threshold.

An analysis of the resulted clusters shows that many of them are small clusters with less than 50 voxels (Fig. 5) and are spatially too compact to portray RFNs. We investigated also the intra-cluster connectivity and measured the number of connections a voxel have with other voxels in a cluster. As shown in Fig. 6, for clusters with less than 50 voxels, the normalized number of connections decreases with the cluster size, while the larger clusters have the opposite trend. Therefore, we choose 50 as the lower limit for clusters to be considered as potential RFNs.

The whole-brain grey matter mask has 13312 voxels and analysis of the ICA results showed that clusters with more than 5000 voxels are too large to be considered as a single coherent RFN and should be refined further. Therefore, we choose 5000 as the ultimate upper limit for a cluster to be considered as an independent RFN. However, it should be pointed out that most of the extracted RFNs were much smaller (see Table 2). By lowering the upper limit, large clusters can be further analyzed with additional iterations to study the hierarchical structures within. This point is further demonstrated by Figure S2 and Appendix C in Supporting Information S1.

Cordes et al. used previously a CC threshold of 0.3 for voxel-based hierarchical clustering [7] and the same threshold was opted here for the individual dataset. Systematically changing the CC threshold showed that increasing the threshold above 0.3 resulted in the loss of robustness of the algorithm. A threshold of CC≥0.4 resulted in only about 0.1% of the values remained.

Regression removal of the global signal was deliberately omitted in the preprocessing of the data, as it is known to introduce substantial negative correlations into the data [12] and lead to controversial interpretation of the resulted RFNs [13]. Without global signal removal, in the averaged CC matrix there are only 0.3% negative values with mean 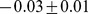. Hence, without the removal of the global signal the vast majority of the negative CC values are too small to be considered statistically significant.

### Comparison between hierarchical clustering and ICA-based methods

**Figure 7:**
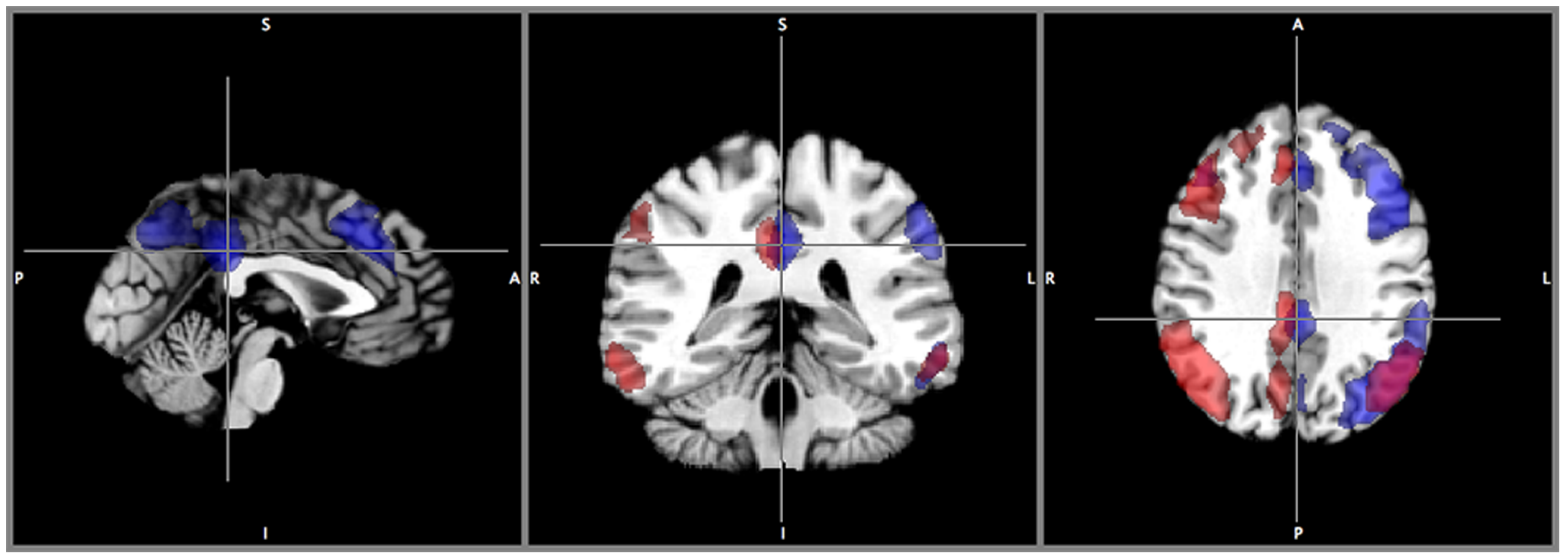
Unilateral networks from ICA analysis. Symmetrical one-sided networks found amongst ICA results. Both in their entirety cannot be obtained through hierarchical clustering due to the spatial overlap between them.

The implemented hierarchical clustering is a deterministic algorithm and the final result is independent of the initial seed point. Its non-fuzziness implies that each voxel is exclusively assigned to a single cluster. Therefore, there is no spatial overlap among the clusters identified by the proposed framework. For example, the two unilateral frontal-parietal networks found with ICA [14] (Fig. 7) are not detectable in their entireties with the proposed hierarchical clustering method, because these two unilateral frontal-parietal networks have an overlapping component in the medial frontal cortex. With hierarchical clustering, detecting one will exclude the other from being detected in its entirety.

The computation involved in hierarchical clustering does not scale up well with the number of observations. Optimal agglomerative algorithms exist for single (SLINK [15]) and complete-linkage (CLINK [16]) scale at Ο(n^2^). Average-linkage algorithms such as the one used here scales cubically. Average-linkage tends to join clusters with small variances and is slightly biased toward producing clusters with the same variance because it considers all members in the cluster rather than just a single point. Hence, average-linkage tends to be less influenced by extreme values than other methods, despite of the fact that hierarchical clustering is overall very sensitive to outliers.

**Figure 8:**
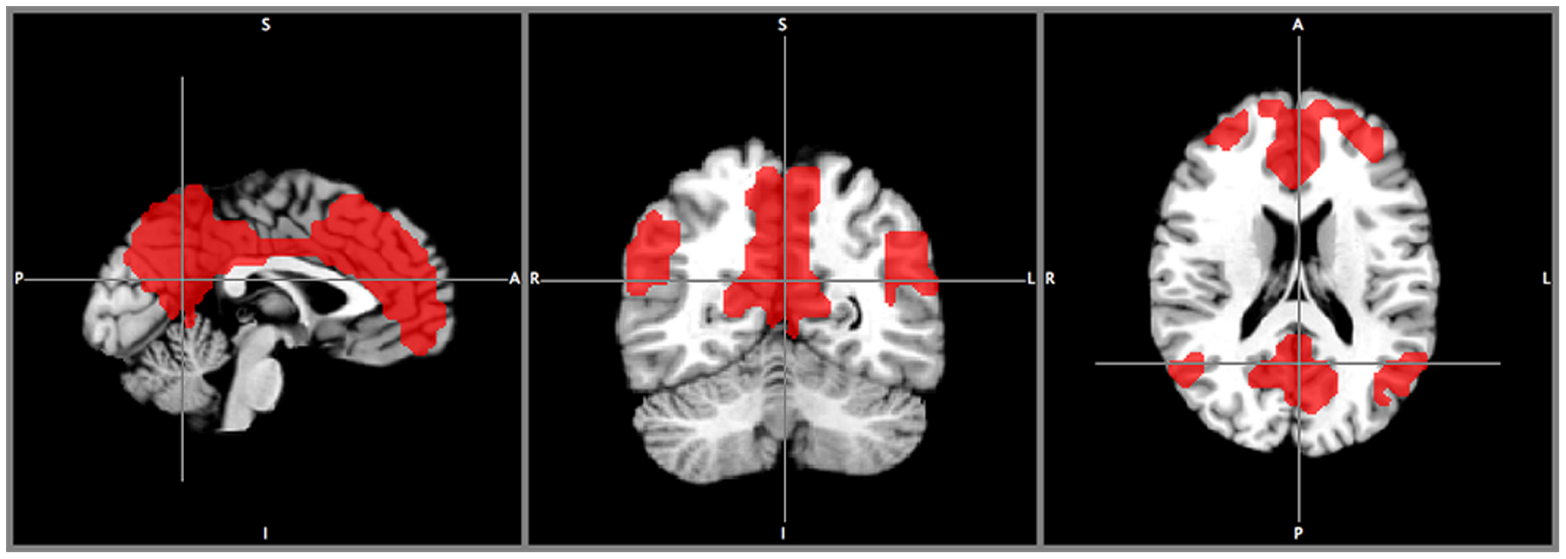
The default mode network. The default mode network in its entirety found at cut number k = 18 from the first iteration. This cluster was found through exhaustive search over all cut numbers.

The used scheme may have not extracted all meaningful clusters from the data, because only a few numbers of cuts are applied to the dendrograms. For example, the DMN was not found in its entirety at the specified cut-levels discussed above. Through exhaustive searching, the whole DMN (Fig. 8) was found amongst clusters at the cut level from the dendrogram of the second iteration.

The DMN result illustrates once again that the hierarchical clustering approach has its inherent strength to reveal the hierarchy structure within a functional connectivity network. As shown in Fig. 3, the DMN in its entirety at cut level k = 18 (Fig. 8) was split into the frontal- (Fig. 3M) and parietal sub-networks (Fig. 3O) down in the dendrogram at cut level k = 32 from the large cluster. It is known that the DMN is composed of the prefrontal and parietal sub-units [17]. The medial prefrontal sub-network is responsible for executive functions and the parietal sub-network is responsible for sensory-related responses.

## Conclusion

With the developed framework we successfully have extracted gray matter clusters with striking similarities to RFNs that are well documented in the literature using different analysis methods. The obtained results further confirm the notion that brain at resting-state is highly engaged in spontaneous synchronous activity within the various intrinsic functional networks. The present study demonstrates also that hierarchical clustering might be a very useful tool for analysis of whole-brain resting-state fMRI data at a voxel-level. This approach is model free and does not require any prior assumption about the number and location of the clusters. Furthermore, it can be used to reveal directly the hierarchical structures within the functional connectivity networks.

## Supporting Information

Figure S1 **Inconsistency coefficients of dendrogram.** Plot of inconsistency coefficients of the full dendrogram. The nodes are sorted from lowest to highest distance in the dendrogram. The coefficients fluctuate sporadically and no general pattern can be detected for determining dendrogram cut level.
(TIF)

Figure S2 **Sub-networks of visual system.** Clustering results from an additional iteration with 8-cluster split of the visual network (Fig. 3R) extracted from the 2^nd^ iteration. The results illustrate the potential for using the proposed framework to study the hierarchical structures within functional connectivity networks. As shown, the visual network was split into a sub-network containing the primary and secondary visual systems (A), the lingual gyrus (B) and inferior temporal gyrus (C). It should be possible to extract the full hierarchical structure tree of the visual system by further analysis of the larger sub-network (A).
(TIF)

Supporting Information S1 **Appendices A, B, and C.**
(DOC)
