# Supplementary figures and images for: Analysis of Whole-Brain Resting-State fMRI Data Using Hierarchical Clustering Approach
Source: PLoS One. 2013 Oct 18;8(10):e76315. doi: 10.1371/journal.pone.0076315 (PMC3799854; doi:10.1371/journal.pone.0076315)

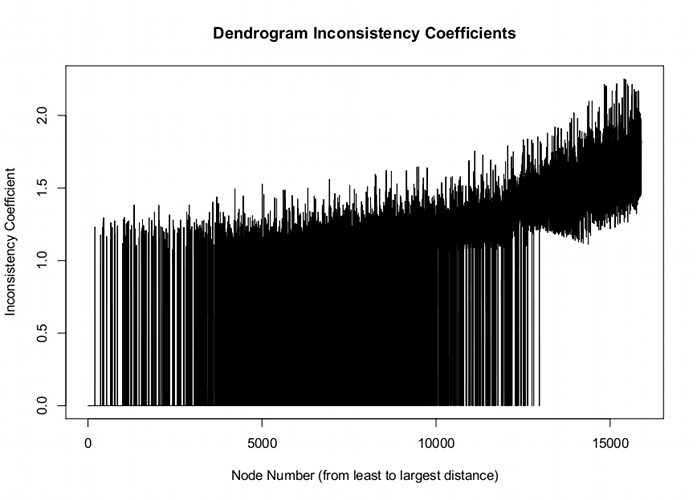

Supplement: Figure S1 — Inconsistency coefficients of dendrogram. Plot of inconsistency coefficients of the full dendrogram. The nodes are sorted from lowest to highest distance in the dendrogram. The coefficients fluctuate sporadically and no general pattern can be detected for determining dendrogram cut level. (TIF) [file pone.0076315.s001.tif]

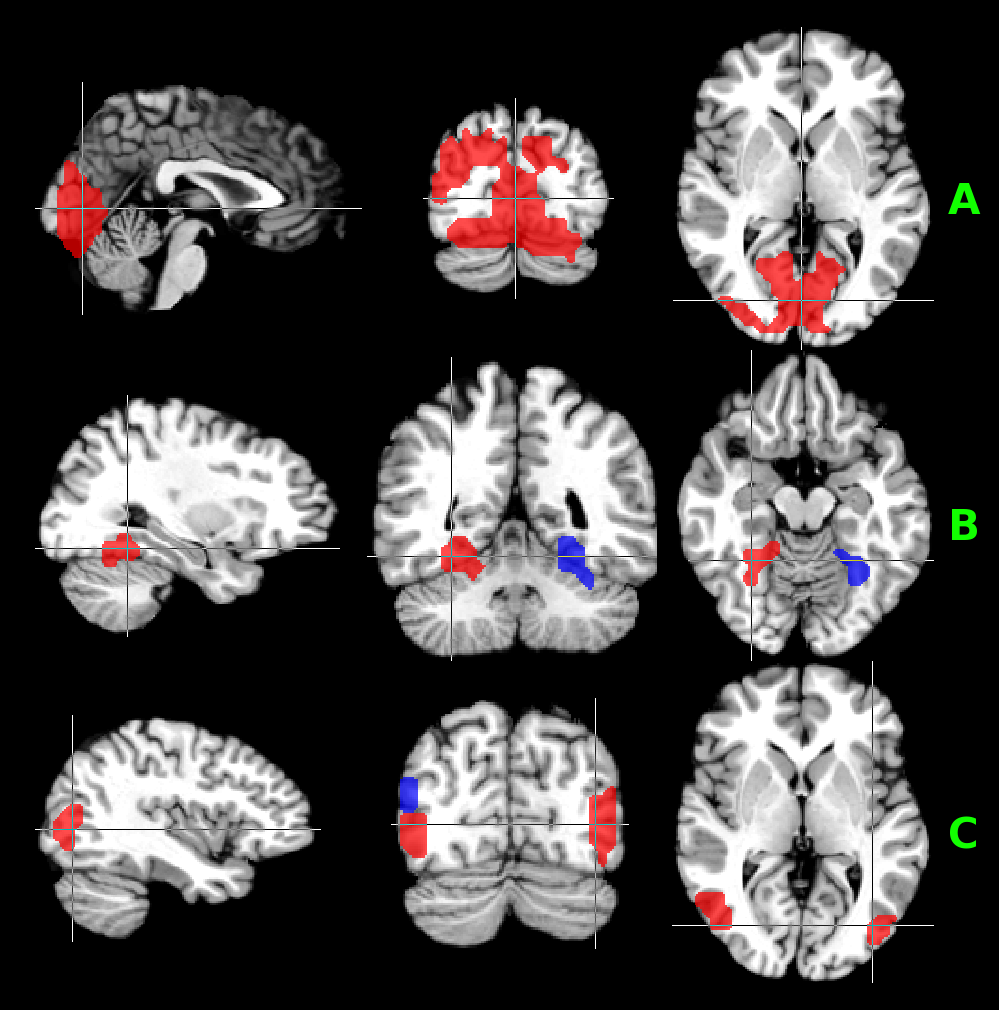

Supplement: Figure S2 — Sub-networks of visual system. Clustering results from an additional iteration with 8-cluster split of the visual network (Fig. 3R) extracted from the 2nd iteration. The results illustrate the potential for using the proposed framework to study the hierarchical structures within functional connectivity networks. As shown, the visual network was split into a sub-network containing the primary and secondary visual systems (A), the lingual gyrus (B) and inferior temporal gyrus (C). It should be possible to extract the full hierarchical structure tree of the visual system by further analysis of the larger sub-network (A). (TIF) [file pone.0076315.s002.tif]
