# Supplementary material for: Analysis of Whole-Brain Resting-State fMRI Data Using Hierarchical Clustering Approach
Source: PLoS One. 2013 Oct 18;8(10):e76315. doi: 10.1371/journal.pone.0076315 (PMC3799854; doi:10.1371/journal.pone.0076315)
Supplement: Supporting Information S1 — Appendices A, B, and C. (DOC) [file pone.0076315.s003.doc]

**Supplementary Information**

**Appendix A: Pseudocode for hierarchical clustering algorithm**

**Input:** Distance matrix, *D*

**For all** *x* in *D* **do**

create_node(*x*)*;*

**end for**

**while** (size(D) > 1) **do**

*minVal* = index_of(min(D));

*clustCol* = *D*[0][*minVal*];

*clustRow = D*[*minVal*][0];

newClust = new_cluster(*clustCol, clustRow*);

connect_new_node(*col_clust, row_clust,* branch_length =

(*min_val/2*));

delete_row(*clustRow* in *D)*;

delete_col(*clustCol* in *D*);

**for all** *x* in *D_copy* **do**

**if (***x* != *clustCol & x* != *clustRow*) **then**

*D*[*newClust*][*x*] = average_linkage(*newClust, x*);

**end if**

**end for**

**end while**

**Appendix B: Dendrogram cut-levels based on inconsistency coefficients**

Dendrogram cut-levels based on inconsistency coefficients (ICs) were investigated. The ICs at full depth for every node in the dendrogram were computed. As seen in Figure S1, the ICs across the nodes vary sporadically and it is difficult to determine a meaningful cut-level criterion based on the ICs without external restraints.

**Appendix C: Hierarchical structure of the Visual Network**

The visual network (Fig. 3R) extracted from the 2^nd^ iteration was further analyzed by using an additional iteration with 8-cluster split. The results provide an illustrative example to demonstrate the potential for using the proposed framework to study the hierarchical structures within functional connectivity networks. As shown, after an additional 8-cluster split, the majority of the visual system is intact as a relatively large cluster (Figure S2-A). This sub-network covers parts of the middle cingulate cortex, calcarine gyrus, and inferior parietal lobe. This sub-network can be considered as a superposition of multiple components of the ICA results reported previously (Allen et al. 2012: Fig. 4-IC46+IC64+IC48). Two clusters covering the bilateral lingual gyrus exhibits good bilateral symmetry (Figure S2-B), representing a visual network similar to Allen et al. 2012: Fig. 4A-IC67. Two clusters occupying the inferior temporal gyrus (Figure S2-C) is reminiscent of Allen et al. 2012: Fig. 4A-IC39. The other 3 clusters were smaller than 50 voxels in size (not shown) and considered as artifacts.
